# The effect of different concentrations and temperatures of sodium hypochlorite irrigation on pain intensity following endodontic treatment of mandibular molars with irreversible pulpitis: A randomized, double‐blind clinical trial

**DOI:** 10.1002/cre2.754

**Published:** 2023-06-22

**Authors:** Hadi Mokhtari, Amin Salem Milani, Vahid Zand, Sahar Shakuie, Leila Nazari

**Affiliations:** ^1^ Department of Endodontic, Faculty of Dentistry Tabriz University of Medical Sciences Tabriz Iran

**Keywords:** cryotherapy, pain intensity, sodium hypochlorite, visual analog scale

## Abstract

**Objectives:**

This study aimed to determine the severity of pain after endodontic treatment of mandibular molars with irreversible pulpitis following the use of sodium hypochlorite (NaOCl) at different temperatures and concentrations.

**Methods:**

In this randomized, controlled clinical trial, 72 patients with mandibular molars with irreversible pulpitis were randomly assigned to six groups. The teeth were anesthetized and the root canals were prepared. During the instrumentation, the root canals were irrigated with NaOCl solution at concentrations of 0.5% and 1% and temperatures of 2.5°C, 22°C, and 40°C, 2.5°C were achieved through cryotherapy. Assessment of pain was conducted before, immediately after, and 3, 24, 48, and 72 h after treatment. After obturation, the patients recorded their pain intensity at different time intervals on the visual analog scale (VAS) and reported the number of analgesics tablets they used. The frequency of analgesics tablets and their effect on pain sensation was recorded in the second part of the form. Repeated measures two‐way analysis of variance test was used to compare the trend of pain changes over time between two intervals of time in each group. Friedman's nonparametric test was used to compare the intragroup mean score of pain over time and Kruskal–Wallis for comparing the intergroup mean score.

**Results:**

Changes in VAS pain scores of all the groups were significant over time (*p* < .001). Pain in all the groups decreased immediately after treatment and increased 3 h after treatment. There were no significant differences in pain ratings and the number of analgesics tablets used in the groups of NaOCl with different concentrations and temperatures over time.

**Conclusions:**

Within the study's limitations, we concluded that there was no significant difference between concentrations of 0.5%, and 1% and temperatures of 2.5°C, 22°C, and 40°C in pain intensity following endodontic treatment of mandibular molars with irreversible pulpitis.

## 1 INTRODUCTION

Sodium hypochlorite (NaOCl) is one of the most effective intracanal irrigants and has antibacterial properties; it dissolves vital and nonvital pulp tissues (Okino et al., 2004). However, its solubility depends on the concentration, volume, and time of contact with the tissue (Moorer & Wesselink, 1982). High concentrations of NaOCl are required to kill acid‐fast bacteria and spores, but there is no consensus on its ideal concentration (Siqueira et al., 2000). Farzaneh et al. (2018) reported more pain at a concentration of 2.5% versus 5.25% NaOCl in patients with irreversible pulpitis treated in one session (Farzaneh et al., 2018). Other studies have found the opposite (Bashetty & Hegde, 2010; Mostafa et al., 2020; Verma et al., 2019).

The disadvantages of NaOCl include toxicity, unpleasant taste, and inability to remove the smear layer due to the lack of effect on minerals (McComb et al., 1976; Spångberg et al., 1973). In addition, NaOCl has toxic effects on periapical tissues, especially at high concentrations, and causes acute inflammation in the area (Ercan et al., 2004; Estrela et al., 2003; Kuruvilla & Kamath, 1998).

Studies have shown that more cytotoxicity occurs with 5.25% NaOCl compared to 1.0% and 0.5% solutions (Chang et al., 2001; Pashley et al., 1985). The main reason for using a low concentration of 0.5% NaOCl instead of 5.25% is the concern over its toxic effects at higher concentrations (Hulsmann & Hahn, 2000). There is consensus that increasing the temperature improves the tissue solubility of sodium hypochlorite, but there are few studies on this (Rossi‐Fedele & De Figueiredo, 2008; Sirtes et al., 2005). Continuous agitation helps 2% NaOCl dissolve soft tissues at the same rate as 6% NaOCl without agitation and increasing the solution temperature from 45°C to 60°C improves the tissue dissolution rate (Stojicic et al., 2010).

Heating low‐concentration solutions improve their tissue solubility without affecting their short‐term stability. Also, the systemic toxicity of solutions is lower compared to solutions with higher concentrations at lower temperatures with the same efficiency. NaOCl solution at 1%, 2.62%, and 5.25% concentrations showed an unusual decrease in chlorine for 1 h at 45°C and 60°C (Sirtes et al., 2005). Also, heating the NaOCl solution increased its ability to dissolve organic matter (Abou‐Rass & Oglesby, 1981; Rossi‐Fedele & De Figueiredo, 2008).

In mandibular molars with symptomatic apical periodontitis, patients undergoing cryotherapy treatment exhibited a significant reduction in postoperative pain levels compared to the control group (Gundogdu & Arslan, 2018).

Vera et al. (2015) showed that using a cold saline solution as the final irrigant reduced the temperature of the outer root surface by 10°C in 4 min in single‐rooted teeth which may be enough to produce a local anti‐inflammatory effect in the periradicular tissues. In teeth with a vital pulp using saline at 2.5°C as the final irrigant can result in a significant reduction in postoperative pain levels (Keskin et al., 2017).

So far, the effect of different concentrations or temperatures of sodium hypochlorite on pain after endodontic treatment has been investigated (Bashetty & Hegde, 2010; Demenech et al., 2021; Farzaneh et al., 2018; KarataŞ et al., 2021; Mostafa et al., 2020; Verma et al., 2019). This randomized, double‐blind clinical trial aimed to determine the severity of pain after the endodontic treatment of mandibular molars with irreversible pulpitis following using NaOCl irrigant at different concentrations and temperatures.

## 2 MATERIALS AND METHODS

### 2.1 Ethical considerations

This study was approved by the Iranian Registry of Clinical Trials (IRCTID: IRCT20220108053665N1). The treatment steps and the process of patient participation in the study were explained to the patients by the researcher. Patients also signed an informed consent form, which included explanations about the study and the patient's voluntary participation. The researcher reassured patients about protecting their personal information, and patients were allowed to leave the study at any time without giving a specific reason.

### 2.2 Inclusion and exclusion criteria

Seventy‐two patients referring to the Endodontics Department of Tabriz Dental School, who met the following inclusion criteria, were randomly selected and evaluated. The inclusion criteria consisted of the following:

– Systemically healthy patients
– Age range 18–65 years
– Lack of sensitivity to epinephrine and sodium hypochlorite
– No facial paresthesia
– Not having taken any painkillers within 12 h before treatment
– Not having taken any medications interfering with anesthesia
– No history of trauma
– No periodontal disease
– Completing an ethical questionnaire and being willing to participate in the study
– Having mandibular molar teeth with symptomatic irreversible pulpitis
  The exclusion criteria included the following:
– Patients not meeting at least one of the inclusion criteria
– Not achieving lip numbness 15 min after block injection
– Pregnancy and lactation
– Severe periodontal disease
– Teeth that could not be isolated with a rubber dam
– Root canal calcification
– Teeth with severe irreparable caries
– Use of a specific drug or medication

### 2.3 Sample and randomization

In selecting the teeth, pulp sensibility tests (cold test, heat test, and electric pulp test) were performed by A.S.M to confirm the diagnosis of irreversible pulpitis. In the cold test, a cold spray containing 1,1,1,2 tetrafluoroethane was sprayed on a large cotton ball (#2) and placed in the middle area of the buccal surface of the mandibular molars (Jones et al., 2002). For electric pulp testing, the probe's tip was placed in the occlusal third of the buccal surface of the tooth and was tested at least twice to confirm the response (Bender et al., 1989). To perform the heat test, the ball burnisher was placed on a hot flame until it turned red and then placed at a distance of 1–2 mm from the buccal surface of the tooth to transfer heat to the tooth (Jafarzadeh & Abbott, 2010). The lower molar teeth that had a sharp and severe response to the cold and heat test, and a positive response to the electric pulp test (EPT) and the pain was lingering (more than 5 min), were included in the study as irreversible pulpitis. To avoid overlap in responses between the pulp sensibility tests, a 2‐min interval was considered.

Based on the results of the study by Mostafa et al. (2020) and estimating the mean and standard deviation of pain in two groups treated with 1.3% and 5.25% concentrations of NaOCl at the rate of 1.25 (±1.66) and 2.94 (±2.16) in their research and taking into account the type 1 error rate of 0.05 and the test power of 90%, the number of patients required for each group was 29. We added 20% to the sample size to increase validity, and we considered 36 patients in each group (36 in the 1% sodium hypochlorite group and 36 in the 0.5% sodium hypochlorite group). To determine the effects of temperature and different concentrations of NaOCl, the subjects were randomly assigned to the following six groups using RandList 1.2 software:

**Group 1**: 1% NaOCl as an antibacterial irrigation solution at room temperature (22°C)

**Group 2**: 1% NaOCl as an antibacterial irrigation solution at 40°C

**Group 3**: 0.5% NaOCl as an antibacterial irrigation solution at room temperature (22°C)

**Group 4**: 0.5% NaOCl as an antibacterial irrigation solution at 40°C

**Group 5**: 1% NaOCl as an antibacterial irrigation solution at 2.5°C

**Group 6**: 0.5% NaOCl as antibacterial irrigation solution at 2.5°C

For each patient, one of the six groups was randomly selected. They were also written on the syringes. The syringes containing the irrigation solutions were covered with a layer of opaque coating. Each patient selected a sheet and based on the selected number, the researcher used one of the solutions for that patient. Researchers and patients did not know the contents of the syringe, and only the person who prepared the solutions and had no role in the clinical process did. Keeping the solutions at the desired temperature was controlled by the person who prepared them without the researcher or patient knowing their contents. Thus, neither the patient nor the researcher was aware of the type of treatment of choice, and the study was conducted as a double‐blind study.

### 2.4 Treatment procedures

NaOCl solutions (1% and 0.5%) were prepared by diluting 5% NaOCl solution (Chloran). To prepare NaOCl solution at 40°C, a 10‐mL syringe was filled with NaOCl solution at room temperature and heated to 40°C using syringe heaters (Alfa). A calibrated electric thermometer (SBG) was used to measure the temperature of each syringe. To prepare NaOCl at 2.5°C, a 10‐mL syringe was filled with NaOCl solution at room temperature and placed in a refrigerator at 2.5°C. A calibrated electric thermometer (SBG) was used to measure the temperature of each syringe. For the blinding purpose, all the steps of NaOCl solution preparation were performed by a trained assistant who had no role in the clinical process. An insulating plastic cover was used on the syringes to prevent the researcher from feeling the temperature of irrigation solutions.

Patients received two cartridges of 2% lidocaine with epinephrine (Darupakhsh) (1:80,000) for the inferior alveolar nerve block before starting root canal treatment. All the treatment steps were performed by A.S.M and after isolation with a rubber dam, followed by preparation of the access cavity, the working length was determined using a Root ZX apex locator (Morita Co.) and radiographically confirmed. In unsuccessful anesthesia, PDL and intrapulpal injections were used as complementary techniques. After preparing the root canals with hand instruments to at least #15 K‐file (Mani), Denco rotary files (Shenzhen Denco Medical) were used to prepare the root canals with a speed of 250–350 rpm and a torque of 1–2 Ncm. Apical patency was checked between each use of the rotary file with a K‐file #10 (Mani), and apical preparation was completed up to the F3 file. During instrumentation, the root canals were irrigated with 2 mL of NaOCl between each successive instrument (in group 1 with 1% solution at 22°C, in group 2 with 1% NaOCl solution at 40°C, in group 3 with 0.5% NaOCl solution at 22°C, in group 4 with 0.5% NaOCl solution at 40°C, in group 5 with 1% NaOCl solution at 2.5°C, and in group 6 with 0.5% NaOCl solution at 2.5°C). A 27‐G needle was inserted into the root canal (Avapezeshk) up to 2 mm shorter than the working length, and the root canals were flushed with upward and downward movements of the syringe with a rubber stop as a guide. The needle was repeatedly moved up and down during irrigation to prevent locking inside the root canals. To prevent NaOCl accidents, side‐vented flushing syringes were used passively and without getting stuck in the root canals. At the end of the root canal preparation process and before obturation, the smear layer was removed from the root canal wall using 3 mL of 17% ethylenediaminetetraacetic acid (Morvabon) and then with 5 mL of normal saline solution.

In the same session, the root canals were dried using paper points (Meta Biomed Co.) and then obturated with gutta‐percha (Meta Biomed Co.) and AH26 sealer (Dentsply Maillefer) using the cold lateral compaction technique. Zonalin (Golchai) temporary restoration was placed. After obturating the root canals, a periapical radiograph was taken for any procedural errors such as underfilling, overfilling, voids, etc. Each patient received a pack containing 10 tablets of ibuprofen (400 mg) (Dana) to take for persistent pain after treatment.

### 2.5 Pain measurement tools

Each patient was assigned a form (consisting of two parts) to complete. The first part consisted of a visual analog scale (VAS) form to record pain intensity from 0 to 10 before and immediately after treatment and 3, 24, 48, 72 h, and 7 days after treatment. The second part of the form was used to record the frequency of analgesics used and their effect on pain sensation. In the second section, the following criteria were used:

Score 0: no pain

Score 1: mild pain that did not require analgesia

Score 2: moderate pain that was controlled with painkillers and did not interfere with sleep or daily activities

Score 3: unbearable pain that was not controlled by analgesia and interfered with daily activities or required incision and drainage.

Patients were asked to contact the clinic if they had severe pain or any questions about treatment.

If the patient developed severe pain or swelling in the area, in an emergency session, examination and treatment with antibiotics, stronger painkillers, or incision and drainage were considered for complete recovery under the researcher's supervision. All the treatment steps were performed by A.S.M.

### 2.6 Statistical analysis

Statistical analyses were performed using SPSS 25. Means and standard deviations of pain intensity scores (VAS from 0 to 10) of patients in NaOCl groups with different concentrations and temperatures were calculated and reported before treatment, immediately after treatment, 3, 24, 48, and 72 h after treatment, and 7 days after treatment. Also, the median and the first and third quartiles of patients' pain scores after analgesia, and the number of analgesics used by patients in NaOCl groups and at different time intervals after treatment were calculated and reported. Due to the normal distribution of VAS pain data, the comparison of changes in pain severity scores in different groups over time was performed by repeated‐measures analysis of variance. Due to the nature of pain score rankings and the number of analgesics used, intergroup comparisons of these variables at each time interval were performed by the Kruskal–Wallis test, and their intragroup comparisons at different times were performed by the Friedman test. Bonferroni tests were used to show the differences between every two‐time points.

## 3 RESULTS

In this study, the mean age of patients was 30.90 years (±7.72) and 62.5% of them were male, and 37.5% were female. In Table 1, the frequency distributions of patients in terms of gender were not statistically significant between the different groups. Also, the mean age of patients and their VAS pain scores before treatment were not significantly different between the study groups.

**Table 1:** Distribution of patients by age, sex, and VAS scores before treatment.

Group variable	0.5% NaOCl, 22°C	0.5% NaOCl, 40°C	0.5% NaOCl, 2.5°C	1% NaOCl, 22°C	1% NaOCl, 2.5°C	1% NaOCl, 40°C	*p* value^a^
Gender							
Male	8 (66.67%)	7 (58.33%)	8 (66.67%)	6 (50%)	9 (75%)	7 (58.33%)	.91
Female	4 (33.33%)	5 (41.67%)	4 (33.33%)	6 (50%)	3 (25%)	5 (41.67%)	
Age	28.42 ± 9.27	31.42 ± 8.99	30.42 ± 6.83	31 ± 6.84	33.67 ± 7.34	30.5 ± 7.44	.72
Preoperative VAS	7.08 ± 1.16	6.75 ± 1.14	7.0 ± 0.95	7.17 ± 1.27	6.83 ± 1.64	6.5 ± 1.38	.81

Abbreviations: ANOVA, analysis of variance; VAS, visual analog scale.

a For qualitative variables Chi‐square test was used and for quantitative variables, the ANOVA test was used.

According to Table 2 and Chart line 1, changes in VAS pain scores were significant in all the groups in intervals of time immediately, 3, 24, 48, 72 h, and 7 days after treatment (*p* < .001). The pain decreased immediately after treatment in all the groups and increased 3 h after treatment. Hourly, the severity of pain decreased up to 7 days after treatment in all the groups. The interactions of time and group were statistically significant (*p* = .028). The rate of pain immediately after treatment in the 0.5% and 1% NaOCl group with a temperature of 40°C was higher than in other groups, but was lower than the others 3 h after treatment. The difference in mean pain severity was not statistically significant between the groups (*p* = .71).

**Table 2:** Means and standard deviations of changes in patients' VAS pain scores over time.

VAS group	Immediately after treatment	3‐h interval	24‐h interval	48‐h interval	72‐h interval	7‐day interval	*p*‐value		
0.5% NaOCl, 22°C	5.5 ± 1.57	6.33 ± 1.23	5.75 ± 1.6	4.0 ± 1.21	2.33 ± 1.67	0.75 ± 1.29	<.001	.028*	.71**
0.5% NaOCl, 40°C	6.0 ± 1.71	6.1 ± 1.6	5.83 ± 1.64	3.92 ± 1.44	2.42 ± 1.83	1.5 ± 1.57	<.001		
0.5% NaOCl, 2.5°C	5.33 ± 1.3	6.25 ± 1.14	4.92 ± 1.44	3.08 ± 1.56	1.83 ± 1.85	1.0 ± 1.35	<.001		
1% NaOCl, 22°C	5.92 ± 1.56	6.33 ± 1.44	5.5 ± 1.57	4.25 ± 1.06	2.58 ± 1.78	1.17 ± 1.11	<.001		
1% NaOCl, 40°C	6.0 ± 1.86	6.3 ± 1.71	5.33 ± 1.87	4.08 ± 1.83	3.42 ± 1.83	1.92 ± 1.68	<.001		
1% NaOCl, 2.5°C	5.0 ± 1.6	6.33 ± 1.87	5.25 ± 2.01	3.17 ± 1.34	1.83 ± 1.7	0.83 ± 1.34	<.001		

Abbreviation: VAS, visual analog scale.

* *p* value is related to interactions of time and group

** *p* value is related to groups.

**Chart line 1:**
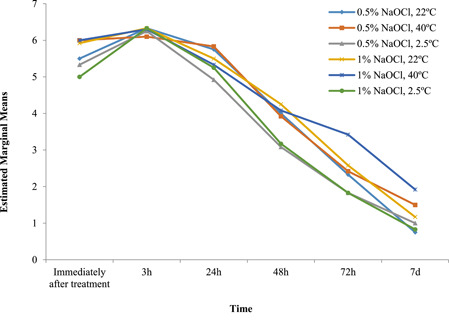
Means and standard deviations of changes in patients' VAS pain scores over time. VAS, visual analog scale.

With Bonferroni test, comparing the times of pairs in Table 3 demonstrated a significant difference between them, with *p* values less than .05.

**Table 3:** Comparison of the studied times two by two.

(i)	(J)	Mean difference (I–J)	Standard error	*p* value^a^ (Bonferroni tests)
VAS score immediately after treatment	VAS score 3 h after treatment	−0.375	0.268	1.000
	VAS score 1 day after treatment	0.194	0.213	1.000
	VAS score 2 days after treatment	1.875	0.203	<0.001
	VAS score 3 days after treatment	3.222	0.231	<0.001
	VAS score 7 days after treatment	4.431	0.213	<0.001
VAS score 3 h after treatment	VAS score 1 day after treatment	0.569	0.184	0.044
	VAS score 2 days after treatment	2.250	0.166	<0.001
	VAS score 3 days after treatment	3.597	0.201	<0.001
	VAS score 7 days after treatment	4.806	0.199	<0.001
VAS score 1 day after treatment	VAS score 2 days after treatment	1.681	0.138	<0.001
	VAS score 3 days after treatment	3.028	0.164	<0.001
	VAS score 7 days after treatment	4.236	0.164	<0.001
VAS score 2 days after treatment	VAS score 3 days after treatment	1.347	0.133	<0.001
	VAS score 7 days after treatment	2.556	0.149	<0.001
VAS score 3 days after treatment	VAS score 7 days after treatment	1.208	0.129	<0.001

Abbreviation: VAS, visual analog scale.

a Bonferroni tests were used to show the differences between every two‐time point.

In Table 4, at each of the evaluation intervals, there was no significant difference between the mean scores of patients, and NaOCl solution at different concentrations and temperatures had the same effects on patients' pain levels.

**Table 4:** Distribution of patients in different groups of irrigation solutions and time intervals after treatment in terms of median and first and third quartiles of pain scores.

Group time	0.5% NaOCl, 22°C	0.5% NaOCl, 40°C	0.5% NaOCl, 2.5°C	1% NaOCl, 22°C	1% NaOCl, 2.5°C	1% NaOCl, 40°C	*p* value^a^ (Kruskal–Wallis test)
Immediately after treatment	(1–1.75) 1	(1–2) 1.5	(1–1.75) 1	(1–2) 1	(1–1.75) 1	(1–2) 1.5	.54
3‐h interval	(1–2) 2	(1–2) 1	(1–2) 2	(1–2) 2	(1–2) 1.5	(1–1.75) 1	.51
24‐h interval	(1–2) 1.5	(1–2) 2	(1–1.75) 1	(1–2) 1	(1–2) 1	(1–2) 1	.6
48‐h interval	(1–1) 1	(1–1) 1	(1–1) 1	(1–1) 1	(1–1) 1	(1–1) 1	.96
72‐h interval	(1–1) 1	(1–1) 1	(0–1) 1	(0.25–1) 1	(0–1) 1	(1–1) 1	.62
7‐day interval	(0–1) 0	(0–1) 1	(0–1) 0	(0–1) 1	(0–1) 0	(0–1) 1	.43
*p* value (Friedman test)^a^	<.001	<.001	<.001	<.001	<.001	<.001	

a Friedman's nonparametric test was used to compare the intragroup mean score of pain over time and Kruskal–Wallis for comparing the intergroup mean score.

According to Table 5, there was no significant difference in the mean number of analgesics taken by patients in each of the irrigants groups, and the concentration and temperature of NaOCl irrigants did not affect the number of analgesics taken by the patients. However, there were significant differences in the mean number of analgesics taken by the patients at the study intervals of time in each irrigation solution group (*p* < .001). The mean number of analgesics taken by the patients immediately after treatment was the least in all the groups.

**Table 5:** Distribution of patients in different groups of irrigation solutions and time intervals after treatment in terms of median and first and third quartiles of analgesics used.

Group time	0.5% NaOCl, 22°C	0.5% NaOCl, 40°C	0.5% NaOCl, 2.5°C	1.0% NaOCl, 22°C	1.0% NaOCl, 2.5°C	1.0% NaOCl, 40°C	*p* value^a^ (Kruskal–Wallis test)
Immediately after treatment	(0–0.75) 0	(0–1) 0.5	(0–0.75) 0	(0–1) 0	(0–0.75) 0	(0–1) 0.5	.54
3‐h interval	(1–1) 1	(0.25–1) 1	(1–1) 1	(1–1) 1	(0–1) 1	(0.25–1) 1	.81
24‐h interval	(1–3) 2	(0.25–3) 3	(1–2.5) 1	(1–3) 1	(0–3.75) 1	(1–3) 1	.81
48‐h interval	(1–3) 2	(0.25–3) 3	(1–2.5) 1	(1–3) 1	(0–3.75) 1	(1–3) 1	.84
72‐h interval	(1–3) 2	(0.25–3) 3	(1–2.5) 1	(1–3) 1	(0–3.75) 1	(1–3) 1	.84
7‐day interval	(1–3) 2	(0.25–3) 3	(1–2.5) 1	(1–3) 1	(0–3.75) 1	(1–3) 1	.84
*p* value^a^ (Friedman test)	<.001	<.001	<.001	<.001	<.001	<.001	

a Friedman's nonparametric test was used to compare the intragroup mean score of pain over time and Kruskal‐Wallis for comparing the intergroup mean score.

As part of this study, we examined the samples on periapical radiographs to determine if overfill had occurred but there was none.

## 4 DISCUSSION

This study aimed to compare the severity of pain after the endodontic treatment in mandibular molars with irreversible pulpitis following the use of NaOCl irrigant at different temperatures and concentrations in a randomized, double‐blind clinical trial.

In the present study, the 10‐cm VAS was used to measure pain, and patients received the necessary training to record their pain scores in the postoperative period (Moher et al., 2010). Also, a 4‐scale pain measure was used to record postanalgesic pain due to its simplicity and convenience (Demenech et al., 2021). Since pain after endodontic treatment is often felt 72 h after treatment (Ng et al., 2004; Pak & White, 2011), there were four‐time intervals of 3, 24, 48, and 72 h were selected, in addition to immediately after treatment, to assess the severity of pain in patients. Also, to compare the research results with other research, a time interval of 7 days were included (Bashetty & Hegde, 2010; Farzaneh et al., 2018).

In this study, changes in VAS pain levels over time were significant in all the groups. According to Table 2, Pain immediately after treatment in the 1% and 0.5% NaOCl groups at 40°C was higher than in other groups; however, 3 h after treatment, it was less severe than in other groups, the difference in mean pain severity between groups was not significant and the effect of temperature and concentration of NaOCl on patients' pain severity after treatment was not significant, but the effect of time was significant. In Table 5, pain scores following analgesia were not significantly different between the NaOCl groups.

NaOCl has dose‐dependent antimicrobial and tissue dissolution effects, which improve at higher concentrations; however, under these conditions, its cytotoxicity increases (Parirokh et al., 2012; Zehnder, 2006). Therefore, the application of lower concentrations of NaOCl is safer.

Farzaneh et al. (2018) reported more pain at a concentration of 2.5% versus 5.25% NaOCl in patients with irreversible pulpitis treated in one session (Farzaneh et al., 2018), in contrast with the results of the present study that the pain intensity was not different in two concentrations of 0.5% and 1%. This difference in results can be due to different concentrations of NaOCl solution. Also, 5.25% NaOCl may dissolve more remaining pulp tissues and therefore can't release inflammatory signaling molecules to induce inflammation and pain. Mostafa et al. (2020) showed that 1% NaOCl, compared to 5.25% NaOCl, resulted in less need for analgesia in mandibular molars in patients with a nonvital pulp at two‐session visits (Mostafa et al., 2020). In the present study, mandibular molars with irreversible pulpitis were evaluated, while Mostafa et al. (2020) evaluated mandibular molars with necrotic pulps.

Verma et al. (2019) reported no significant differences in the incidence and severity of pain with 5% and 1% NaOCl concentrations after initial endodontic treatments, although lower degrees of pain were recorded in the low‐concentration group (Verma et al., 2019), consistent with the present study. However, different concentrations of NaOCl have been evaluated in these studies.

Flare‐up pain is associated with pulp necrosis, symptomatic apical periodontitis, and periapical radiolucency after treatment (Walton, 2002). Lack of apical constriction causes more material to escape into the preapical region (Cemaltinaz et al., 2005). At the same time, healthy periapical tissues act as a barrier against detergents and material leakage (Psimma et al., 2013). Teeth with pulp necrosis are also more likely to cause irrigation solutions to leaking into the periapical region than teeth with a vital pulp (Salzgeber & Brilliant, 1977). This study was performed on vital teeth with irreversible pulpitis and without periapical pathosis. No overfilled root canal treatments were observed, probably as a result of healthy periapical tissues.

According to Table 2 in the present study, in all the groups, the severity of pain decreased immediately after treatment and increased 3 h after treatment; after 3 h, the severity of pain in all the groups decreased up to 7 days after treatment, indicating significant changes in pain severity during the study period. Reduction of pain severity immediately after treatment may be associated with persistent effects of local anesthesia. The findings of the present study are consistent with previous studies in terms of decreasing the severity of pain 48–72 h after treatment (Bashetty & Hegde, 2010; Farzaneh et al., 2018; Pak & White, 2011).

Gundogdu and Arslan (2018) showed that in mandibular molars and mandibular teeth with symptomatic apical periodontitis, patients undergoing cryotherapy treatment exhibited a significant reduction in postoperative pain levels compared to the control group (Gundogdu & Arslan, 2018). The results of this study are in contrast with the present study, in which there was no difference in the pain intensity of patients with NaOCl solution at temperatures of 2.5°C, 22°C, and 40°C in Table 2. This difference in the results can be due to the different pulp and periapical conditions of the teeth. The present study evaluated mandibular molars with irreversible pulpitis and normal periapical tissue, while Gundogdu and Arslan evaluated teeth with symptomatic apical periodontitis. However, in Table 2 at 48‐ and 72‐h intervals, temperatures of 2.5°C caused relatively less pain in patients than at other temperatures and the same concentrations of sodium hypochlorite, but these differences were not significant.

Keskin et al. (2017) evaluated the pain intensity after root canal treatment in teeth with a vital pulp using saline at 2.5°C and showed a significant reduction in pain with cryotherapy (Keskin et al., 2017), which is different from the results of the present study, possibly due to different pulpal conditions of the teeth and the type of irrigation solutions. These studies have evaluated normal saline solution at different temperatures, but the present study was performed on NaOCl at 2.5°C, 22°C, and 40°C temperatures. On the other hand, increasing the temperature improves the disinfection ability of NaOCl at low concentrations (Macedo et al., 2017). In the present study, 2.5°C, 22°C, and 40°C temperatures of NaOCl were evaluated. An in vitro study showed that 2.5°C normal saline reduced the temperature of the outer root surface by 10°C in 4 min (Vera et al., 2015). Differences between different studies in pain relief effects using NaOCl at high and low temperatures can be explained by the different temperatures studied.

Overfilling material is associated with more pain following endodontic treatment, according to Demenech et al. (2021). Our study found no overfilling in the patients' periapical radiographic evaluation, it was conducted in the postgraduate department, which may explain the high quality of the root treatments.

Different preoperative and intraoperative variables (e.g., age, sex, tooth type, the severity of pain, number of visits, type of the irrigation solution, canal preparation technique, sealer type and the analgesic used) have been shown to influence the pain severity following endodontic treatments (Arias et al., 2013; Nagendrababu & Gutmann, 2017). They did have no impact on the results of the present study because of the even distribution of these variables between study groups. In this study, to prevent bias due to the presence of preoperative pain, only patients with symptomatic irreversible pulpitis were included, and to reduce the effect of analgesics used before treatment on the severity of pain after the treatment, not taking analgesics 12 h before treatment was one of the inclusion criteria. The mandibular molars were selected because these teeth have complex anatomy, isthmuses, and lateral canals and are difficult to clean and prepare, have a low rate of periapical healing, and exhibit the worst pain scenario after treatment (Arias et al., 2013; Ng et al., 2004).

One of the limitations of this study was the small size of it and only 12 patients were excluded from this, but we conducted this study based on the time we had and the patients who met the inclusion and exclusion criteria. We hope that studies with more participating patients will be conducted in this field in the future. In this study, in unsuccessful anesthesia, PDL and intrapulpal injections were used as complementary techniques, which is likely to reduce posttreatment pain in these patients. This study used low NaOCl concentrations to reduce cytotoxicity, which may contribute to the lack of pain difference between groups. It would be better to study other concentrations in future studies to determine its effect on pain. Our study selected patients who did not take any painkillers 12 h before the treatment, despite most of them having moderate pain before the treatment. It would be more useful to examine the effects of painkillers and not taking painkillers before the treatment on posttreatment pain in future studies.

## 5 CONCLUSION

Within the limitations of the present study, we concluded that there was no significant difference between concentrations of 0.5% and 1% and temperatures of 2.5°C, 22°C, and 40°C in pain intensity following endodontic treatment of mandibular molars with irreversible pulpitis.

## AUTHOR CONTRIBUTIONS

*Study conception, design, proposal writing, and data collection*: all authors. *Analyzing the data and drafting the manuscript*: Hadi Mokhtari and Amin Salem Milani. *Revised manuscript*: Leila Nazari. *Final version of the manuscript*: Vahid Zand and Sahar Shakouie. *The approval of submission of the manuscript*: all authors.

## CONFLICT OF INTEREST STATEMENT

The authors declare no conflicts of interest.

## Data Availability

The data supporting this study's findings are available on request from the corresponding author.
